# Age-Related Effects on Cognitive-Locomotor Dual-Task Abilities in Activities Representative of Daily Life Among Young Seniors

**DOI:** 10.1177/00315125251332325

**Published:** 2025-04-11

**Authors:** Anne Deblock-Bellamy, Anouk Lamontagne, Bradford J. McFadyen, Marie-Christine Ouellet, Andreanne K. Blanchette

**Affiliations:** 1 Occupational Therapy Department, 111832 University of Applied Sciences of Western Switzerland (HETSL|HES-SO), Lausanne, Switzerland; 2 School of Physical and Occupational Therapy, 5620 McGill University, Montreal, QC, Canada; 3 459878 Jewish Rehabilitation Hospital - CISSS Laval Research site of the Centre for Interdisciplinary Research in Rehabilitation of Greater Montreal (CRIR), Laval, QC, Canada; 4 School of Rehabilitation Sciences, 4440 Université Laval, Quebec City, QC, Canada; 5 560498 Center for Interdisciplinary Research in Rehabilitation and Social Integration (Cirris) - CIUSSS de la Capitale-Nationale, Quebec City, QC, Canada; 6 School of Psychology, 4440 Université Laval, Quebec City, QC, Canada

**Keywords:** age-related effects, dual-task, locomotion, cognition and virtual-reality

## Abstract

**Objective:** This study examined whether dual-task (DT) cognitive-locomotor interferences are present among young seniors (55–75 years) simultaneously performing a locomotor and a cognitive task of varying levels of complexity while ambulating in a virtual community environment. **Method:** To assess DT abilities, participants were asked to walk down a virtual mall corridor while remembering a 5-item shopping list. Two levels of complexity for the locomotor (without vs. with obstacles) and the cognitive task (unmodified vs. modified shopping list) were assessed. After measuring the presence of locomotor and cognitive DT costs (DTC) using one sample Wilcoxon signed-rank tests, a nonparametric ANOVA was performed to explore the impact of task complexity on DTC. Spearman coefficients were used to examine the impact of age on DTC. **Results:** Sixteen participants were recruited. Locomotor and cognitive DTC were observed in all DT conditions, except the easiest combination (no obstacle + unmodified shopping list). These DTC were mainly impacted by the complexity of the cognitive task. They were also positively correlated to age. **Discussion:** The results highlight the importance of real-life scenarios for accurately describing DT abilities for whom locomotor DTC seems to increase with age despite the absence of daily limitations.

## Introduction

Aging is a natural process characterized by subtle and progressive alterations in anatomical structures and physiological functions. Common manifestations of aging include various visual changes (Erdinest et al., 2021), a decline in memory and processing speed (Sánchez-Izquierdo & Fernández-Ballesteros, 2021), and reduced walking speed (Pirker & Katzenschlager, 2017). It is estimated that 5%–13% of individuals aged 60 to 70 have lower muscle mass with poorer physical performance and this prevalence increases to 11%–50% for those aged 80 or above (von Haehling et al., 2010). Furthermore, around the age of 60, aging may be accompanied by a decrease in cortical volume, thickness, and surface area (Zhao et al., 2019).

All these changes lead to an overall functional decline highlighted in several studies comparing physical and cognitive capacities between young and older adults (Bohannon & Williams Andrews, 2011; Menz et al., 2004; Nóbrega-Sousa et al., 2020). According to numerous studies, the functional changes attributed to natural aging tend to become more prominent typically after the age of 70 or 80 years. For instance, the prevalence of gait disorders among community-dwelling elderly increases significantly between individuals aged 60–69 years (10.7%) and individuals aged over 80 years (61.7%; Mahlknecht et al., 2013). Cognitive abilities, such as executive functions, and language proficiency seemed also to decline after 70 years old (Harada et al., 2013; Nóbrega-Sousa et al., 2020; Zanto & Gazzaley, 2019). Individuals aged over 85 are less independent to perform activities of daily living than younger elderly (Carmona-Torres et al., 2019). Thus, it seems that younger seniors, around 60 years old, are in a transition period in which anatomical and physiological changes have already begun but behavioral changes are not yet seen. This observation could be explained by compensatory mechanisms which allow younger elderly individuals to compensate for those anatomical and physiological changes and, consequently, delayed the onset of behavioral changes. As an example, younger elderly individuals gradually compensate the loss of gait automaticity by increasing demands on their attentional resources to maintain their locomotor performance (Nóbrega-Sousa et al., 2020).

Young seniors seem to encounter no more significant challenges in executing basic daily activities compared to young adults. Nonetheless, their capability to undertake complex activities as part of their daily routine warrants exploration. In everyday life, it is common to perform another task while walking in the community, such as talking, texting, or planning the purchases for our next meal. Engaging in dual-task (DT) activities, such as talking while walking, can affect the performance of either or both tasks. In a dual-task scenario, maintaining the same level of efficiency as when tasks are executed separately appears challenging (Al-Yahya et al., 2011; Plummer & Eskes, 2015; Plummer et al., 2015). Different neuropsychological theories postulate that these DT interferences reflect competitions in limited processing resources solicited at the same time (Pashler, 1994b; Tombu & Jolicoeur, 2005). Some studies did not observe age-related decline in cognitive-locomotor performance in DT before the age of 70 (Hausdorff et al., 2008; Nóbrega-Sousa et al., 2020). However, these studies have exposed participants to DT conditions with low level of complexity, which may not reflect real-life locomotor DT activities. Indeed, most cognitive-motor DT assessments include cognitive tasks such as backward counting, substitutions, walking trail making performed during forward walking over distance ranging from 3 to 30 m (Bishnoi & Hernandez, 2021; Hausdorff et al., 2008; Hennah et al., 2021). As a result, it remains difficult to accurately estimate the impact of DT ability on everyday life of the elderly, based solely on these findings.

To the best of our knowledge, there is very little evidence on DT ability of healthy young senior during activities representative of daily living, especially in this transitional period characterized by the onset physiological changes without significant observable behavioral changes. The objectives of the present study are therefore to determine if DT cognitive-locomotor interferences are present among younger seniors during DT walking activities representative of daily living and to explore the effects of task complexity and age among young elderly on locomotor and cognitive DT abilities within this group.

## Methods

### Participants

The sample size was calculated using an a priori power analysis based on data from a previous study that followed the same protocol (Deblock-Bellamy et al., 2022). In this study, significant dual-task costs (DTC) were observed in healthy older participants with an effect size greater than 1.0 during the most representative daily dual-task condition. In the present study, we used a more conservative effect size of 0.8 to calculate the sample size, with 80% power (*p* < .05, Wilcoxon signed-rank test, two-tailed). This analysis yielded a minimum sample size of 15 participants. To account for possible drop-outs or technical issues, 16 participants were recruited.

Healthy adults aged 55 to 75 were recruited through a convenience sampling method using Université Laval’s mailing list. Participants were excluded if they reported a neurological condition or locomotor deficits that could affect performance during the experiment. They were also excluded if they had cognitive impairments, determined by a score <26 on the Montreal Cognitive Assessment (MoCA; Nasreddine et al., 2005). Prior to their participation in the study, all participants read and signed a consent form. This study was approved by the institutional ethics review board of the Centre intégré universitaire de santé et de services sociaux de la Capitale-Nationale (CIUSSS-CN; # 2019-1720).

### Outcome Measures and Experimental Protocol

In this cross-sectional study, participants took part in two assessment sessions at Centre for Interdisciplinary Research in Rehabilitation and Social Integration (Cirris) of the CIUSSS-CN. During the first session, participant eligibility was validated, and sociodemographic as well as clinical data were collected. Subsequently, in the second session, DT abilities during activities representative of daily living were assessed.

In the first session, participants completed sociodemographic (age, gender, occupation, and leisure activities) and general health (history of neurological and/or musculoskeletal disorders) questionnaires. Thereafter, an experienced rehabilitation professional conducted clinical assessments to characterize their balance, as well as locomotor and cognitive functions. Cognitive function was assessed with the MoCA, which was administered to all participants by a research team member with a MoCA certification. Walking speed (both preferred and maximal) and endurance were assessed using the *10-m Walk Test* (10MWT; Bohannon, 1997) and the *6-min Walk Test* (6MWT; Bohannon, 2007), respectively. A mean of two trials was calculated for each walking speed measurement. Balance was evaluated using the *Mini Balance Evaluation Systems Test* (Mini BESTest; O’Hoski et al., 2014). Moreover, participants self-reported their confidence in performing various activities without losing balance or experiencing unsteadiness using the *Activities-Specific Balance Confidence Scale* (ABC Scale; Salbach et al., 2006).

**Figure 1.**
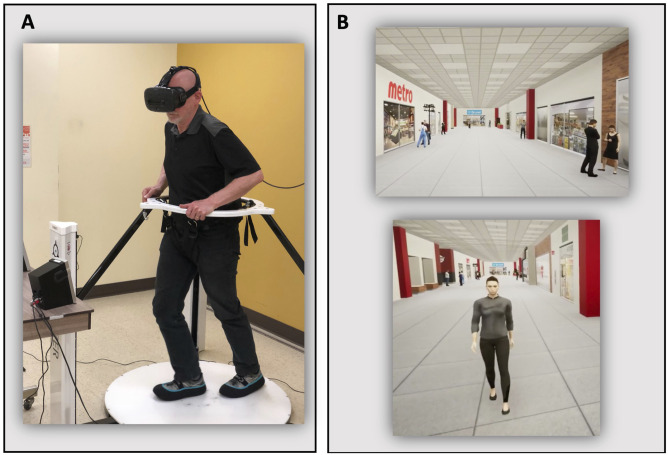
Experimental setup. (A). Omnidirectional platform (Virtualizer, Cyberith GmbH) and virtual reality headset (Vive, HTC); (B). Virtual shopping mall corridor without (upper panel) or with virtual agent avoidances (lower panel).

To assess locomotor and cognitive DT abilities within a standard, safe and representative environment of daily living (second session), our research team previously developed a virtual-reality (VR) based dual-task assessment protocol. This protocol has been employed in healthy young adults and stroke survivors (Deblock-Bellamy et al., 2021, 2022). In DT conditions, participants were placed on an omnidirectional platform and instructed to walk along a virtual mall corridor (Virtualizer, Cyberith GmbH, Vienna, Austria) while remembering a 5-item shopping list. Participants were immersed in this virtual environment (VE) using a VR headset (Vive, HTC Corporation). The omnidirectional platform is composed of a low-friction baseplate and a rotatable ring at the pelvic level. Participants can thus walk in any direction in the VR environment, while remaining on the platform in the physical environment (Cakmak & Hager, 2014). Six optical motion sensors embedded in the platform tracked foot movements and controlled the translation of the participant in the VE, while an optical motion sensor, embedded in a ring attached to the pelvis through a harness, controlled the rotational orientation. Together, these sensors enabled the participant to progress in all directions (360°; in horizontal plane) of the VE. The headset motion sensors tracked head orientation in relation to the participant’s platform orientation and translation within the VE. The tight-fitting harness fixed to the platform ring along with mechanical stops in the vertical direction, served to prevent falls on the platform, although it did not offer weight support. Participants had the option to lean lightly on the ring with their hands, if necessary (Figure 1).

During all DT experimental conditions, participants were immersed in a 75-m virtual straight shopping mall corridor, created in Unreal Engine 4.19.2 (Epic Game, Cary, North Carolina, USA). The VE featured virtual agents, both mobile and stationary, that did not interfere with the participants’ walking trajectories (visual distractors only). Additionally, ambient noises typically found in shopping malls were played through speakers positioned within 1 m of the participant. Two different levels of complexity in locomotor and cognitive tasks were used to assess DT abilities (Table 1). For the simple locomotor task, participants had to walk forward along the corridor. The complex locomotor task included the avoidance of 3 virtual agents on this trajectory. The simple cognitive task was a memory task consisting of 5 shopping items with a free oral recall. The complex cognitive task required a modification of 2 items in the shopping list halfway through the trial. Cognitive ST were performed while participants were seated, both in the physical and virtual environments. More details about the tasks can be found in Deblock-Bellamy et al. (2021).

**Table 1.** Description of Tasks (Locomotor and Cognitive) and Performance Outcomes.

	Level of Complexity	Tasks	Performance Outcomes
Locomotor task	Simple	75-m forward walking	Walking speed
	Complex	75-m forward walking with 3 virtual agents to avoid	
Cognitive task	Simple	Memorization of a 5-item shopping list	Number of correct items remembered
	Complex	Memorization of a 5-item shopping list with modification of 2 items at middle course	

Participants performed locomotor and cognitive tasks separately (ST) and simultaneously (DT) in the VE. The 4 ST and the 4 DT combinations (total of 8 experimental conditions) were performed following a pseudo-random sequence. Each experimental condition was repeated three times (i.e. 3 blocks) for a total of 24 trials. Cognitive ST retention duration was established based on the duration of the DT condition involving the same cognitive task. For this reason, cognitive ST were thus conducted at the end of each block. Participants were not given any information about the condition (ST or DT) or the level of difficulty of the tasks before the trial. No instruction about prioritization was provided.

To quantify locomotor performance, walking speed was used. Cognitive accuracy was characterized with the number of items correctly recalled. For each dependent variable, dual-task costs (DTC) were calculated using the mean of 3 trials, with the following formula (Li et al., 2018; Plummer-D’Amato et al., 2012):

$$\frac{\left(\text{mean}\;ST\;perfomance\right)-\left(\text{mean}\;DT\;perfomance\right)}{mean\;ST\;perfomance}*100$$

Positive DTC for walking speed and cognitive accuracy indicates a decrease in speed and in the number of correctly remembered items during DT compared to ST.

At the end of the experiment, participants were asked to complete two questionnaires measuring the level of cybersickness (French version from the Cyberpsychology Lab of Université du Québec en Outaouais [UQO], 2013; based on Kennedy et al., 1993) and the level of presence in the VE (Presence Questionnaire; Cyberpsychology Lab of UQO, 2002; based on Witmer & Singer, 1998). The Simulator Sickness Questionnaire (SSQ) documented 16 VR side effects using a 4-point scale (0 = none; 1 = slight; 2 = moderate; 3 = severe). Cybersickness was quantified with an average score for each participant. The lower the score, the less effect was observed. The Presence Questionnaire comprised 22 items assessed with a 7-point scale. A higher score on the questionnaire indicates a stronger feeling of presence. An average score was calculated to quantify the feeling of presence for each participant.

Data processing was performed using custom-made scripts written in MATLAB R2018b (The MathWorks, Inc, Natick, MA, USA) and Microsoft Excel 16.24 (Microsoft Corporation, Redmond, WA, USA).

### Statistical Analyses

Nonparametric statistics were used to describe participants (sociodemographic and clinical data), as well as DTC. To determine if DTC medians were different from zero, one sample Wilcoxon signed-rank test was performed (**Obj. 1**). Moreover, a nonparametric two-way repeated measure ANOVA in factorial experiments (ANOVA-type statistic - ATS, Nonparametric Analysis of Longitudinal Data in Factorial Experiments – nparLD) was used to compare all DTC measured across all DT conditions. If significant interactions were observed, post-hoc analyses were planned to use a one-way nparLD (**Obj. 2**). Spearman coefficients with one-tailed *p*-values were performed to examine whether older participants had greater DTC (**Obj. 3**). For all other statistical tests, significance level was set at 0.05.

Descriptive statistics were performed using IBM SPSS Statistics 28.0 (SPSS Inc, Chicago, IL, USA). The nparLD package was used to perform nonparametric repeated measure ANOVA in R software (R Studio 1.2, Inc, USA).

## Results

### Participants

Sixteen participants aged 55 to 75 were recruited (9 females). No locomotor or cognitive impairments were highlighted in the participants for their age group, as indicated by the clinical outcome measures (Table 2), except for one participant whose Mini BESTest score fell slightly below the age-related standards (24 pts with a norm of 26.1). All participants had at least 12 years of formal education.

**Table 2.** Sociodemographic and Clinical Outcome Measures.

	Minimum	25^th^ Percentile	Median	75^th^ Percentile	Maximum
Age (years)	56.00	60.50	**64.00**	69.75	74.00
Gait function					
10MWT preferred speed (m/s)	1.16	1.38	**1.47**	1.59	1.94
10MWT maximal speed (m/s)	1.68	1.87	**1.99**	2.16	2.36
6MWT (m)	477.00	572.50	**604.00**	666.25	777.00
Balance function					
Mini BESTest (max: 28 pts)	22.00	24.25	**25.00**	26.75	28.00
ABC scale (%)	89.06	94.54	**96.60**	99.07	100.00
Cognitive function					
MoCA (max: 30 pts)	27.00	27.00	**28.50**	29.75	30.00
VR-related questionnaires					
Simulator sickness questionnaire (max: 3 pts)	0.00	0.08	**0.25**	0.36	0.94
Presence questionnaire (max: 7 pts)	2.91	4.84	**5.39**	5.82	6.41

In general, the VR-based protocol was well tolerated with a SSQ median score of 0.25/3 (25^th^; 75^th^ percentiles: 0.08; 0.36). Feeling of presence median was 5.39 (4.84; 5.82) out of a total score of 7.

Three participants did not complete the entire VR-based DT assessment protocol (i.e., 24 trials). One participant experienced cybersickness (SSQ mean score of 0.94/3), while the other two reported physical fatigue as the reason for discontinuing the assessment. However, all three performed at least one repetition of all experimental conditions.

### Locomotor and Cognitive DTC in Activities with Different Levels of Complexity

Locomotor and cognitive DTC are presented in Table 3. When participants were exposed to the simple locomotor and cognitive tasks, no DTC has been observed. When participants were required to execute a complex locomotor task paired with a simple cognitive task, only cognitive accuracy DTC (7.14%, *p* = .006) was found. In all conditions proposing the complex cognitive task, DTC were observed on cognitive and locomotor variables, irrespective of the locomotor task complexity (5.40%–22.26%, *p*-values from .017 to < .001). These results indicate that participants walked more slowly when asked to take into consideration changes in the item list while walking, irrespective of the specific locomotor task performed. Additionally, they demonstrated a higher frequency of errors in recalling items from the list compared to ST conditions.

**Table 3.** DTC Median (25^th^–75^th^ Percentiles) for Each DT Condition.

			Cognitive Tasks			
			5-Item List		5-Item list with Modifications	
			DTC	*p*-Value*	DTC	*p*-Value*
Locomotor tasks	Forward walking	Walking speed	3.22 % (−1.61; 10.15)	.079	5.40 % (2.08; 10.83)	**.017**
		Cognitive accuracy	6.67 % (0.00; 12.50)	.059	19.05 % (7.85; 35.99)	**.005**
	Forward walking with virtual agents	Walking speed	−0.25 % (−4.72; 6.38)	.756	7.86 % (4.32; 11.29)	**.002**
		Cognitive accuracy	7.14 % (1.67; 13.50)	**.006**	22.26% (12.95; 30.22)	**< .001**

Significant *p*-values (*p* < .05) are in bold; *: Wilcoxon signed-rank tests.

The impact of locomotor and cognitive task complexity on DTC was investigated (Table 4). Significant interaction between the level of complexity in locomotor and cognitive tasks was found for walking speed DTC. Post-hoc tests were conducted to compare the walking speed DTC in the four experimental conditions. The results indicated that under complex locomotor task conditions, the DTC for walking speed was significantly higher when participants performed a complex cognitive task compared to a simple cognitive task (*p* < .001). No significant differences were observed for the other comparisons (*p-values* from .110 to .382). No significant interaction between the complexity levels of locomotor and cognitive tasks was demonstrated for cognitive accuracy DTC. Nevertheless, significant main effects emerged concerning the complexity of the cognitive and locomotor tasks in cognitive accuracy DTC. Main effect related to complexity of cognitive task indicated that, irrespective of the locomotor task complexity, cognitive accuracy DTC were greater when participants encountered a more complex cognitive condition (*p* < .001). Similarly, regardless of the cognitive task performed, participants exhibited greater cognitive accuracy DTC during the more complex locomotor condition (*p* = .049).

**Table 4.** Effects of Locomotor and Cognitive Task Complexity on DTC.

	Tasks	Median (25^th^–75^th^ Percentiles)	Main Effect		Interaction
			Locomotor Task Complexity	Cognitive Task Complexity	Locomotor Task Complexity*Cognitive Task Complexity
Walking speed (WS) DTC	Forward walking	3.22 % (−1.61; 10.15)	ATS = .05 *p* = .823	ATS = 9.784 ***p* = .002**	ATS = 7.165 ***p* = .007**
	5-Item list				
	Forward walking	5.40 % (2.08; 10.83)			
	5-Item list with modification				
	Forward walking with virtual agent	−0.25 % (−4.72; 6.38)			
	5-Item list				
	Forward walking with virtual agent 5-item list with modification	7.86 % (4.32; 11.29)			
Cognitive accuracy DTC	Forward walking	6.67 % (0.00; 12.50)	ATS = 3.87 ***p* = .049**	ATS = 17.126 ***p <* .001**	ATS = 0.069 *p* = .792
	5-Item list				
	Forward walking	19.05 % (7.85; 35.99)			
	5-Item list with modification				
	Forward walking with virtual agent	7.14 % (1.67; 13.50)			
	5-Item list				
	Forward walking with virtual agent 5-item list with modification	22.26% (12.95; 30.22)			

Significant *p*-values are in bold; ATS: ANOVA-type statistic; WS: walking speed.

### Age-Related Effects on Locomotor and Cognitive DTC

**Figure 2.**
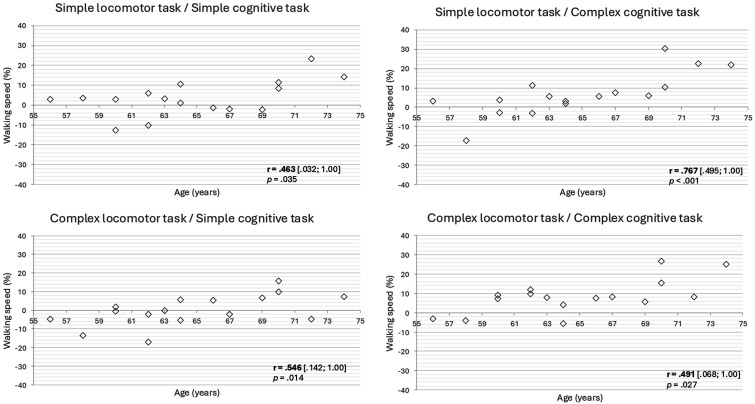
Relationships between age and walking speed DTC in all DT conditions. r = Spearman coefficients [95% confidence intervals].

Significant positive correlations were observed between age and walking speed DTC (*r*: .463 to .767; *p*-values from .035 to <.001) in all DT conditions (Figure 2). These significant correlations indicated that the older the participants, the greater DTC in walking speed. No significant age effect was found on cognitive accuracy DTC in all experimental conditions (*p*-values from .131 to .394).

## Discussion

The present study has explored DT locomotor and cognitive abilities among healthy young seniors using a VR-based assessment protocol that involved activities representative of everyday life. DTC in cognitive and/or locomotor performances were observed in all experimental conditions, except the simplest one. These DTC were notably influenced by the complexity levels of both the locomotor and cognitive tasks. Finally, this study had revealed an age-related effect on locomotor DTC, indicating that among young seniors, older participants exhibited higher walking speed DTC. However, this relationship was not observed in cognitive DTC.

### Locomotor and Cognitive DTC in Healthy Young Seniors

When individuals engage in walking while simultaneously performing a cognitive task, various DT interference patterns can arise, including gait interference (DT-related change only in gait performance), cognitive interference (DT-related change only in cognitive performance), mutual interference (DT-related change in both motor and cognitive performances) or no interference (no DT related change in both performance; Plummer & Eskes, 2015). In the present study, three DT interference patterns were observed, varying across conditions. Specifically, when participants executed a combination of simple locomotor and simple cognitive tasks, no interference was shown, while cognitive interferences or mutual interferences were observed in the other DT conditions. Based on neurophysiological theories (Pashler, 1994a; Tombu & Jolicoeur, 2005), these findings suggest that healthy young seniors may reach the limits of their attentional resources when engaged in DT activities. The inability to sustain performance levels observed in ST scenarios during DT activities reflects the overload on attentional resources. Indeed, participants showed poorer cognitive accuracy when instructed to memorize a list of 5 items while walking and avoiding the virtual agents compared to ST. This observation might indicate that healthy young seniors had prioritized locomotor rather than cognitive performance in this DT condition. The prioritization of gait over cognitive performance seems to be a strategy adopted by individuals to cope with age-related changes. To ensure their safety, individuals may have paid more attention on maintaining postural stability while walking than on the cognitive task (Yogev-Seligmann et al., 2012). The same pattern of DT interference has been observed when community-dwelling seniors were exposed to a similar DT condition in the physical world, i.e. a simple cognitive task combined with a complex locomotor task (Schrodt et al., 2004). In the latter study, participants completed a memorization task while walking at their fastest speed along a 10 m walkway and step over an obstacle. The execution of the locomotor task thus seems to be prioritized over the cognitive task by older participants. In the present study, when participants had to perform cognitive complex task, mutual interference emerged, regardless of the difficulty of the locomotor task performed simultaneously. Mutual interference while performing complex cognitive task (e.g. Stroop Test) has been already observed in older individuals (Plummer-D’Amato et al., 2011, 2012; Siu et al., 2008). Taken together, these results suggest that DT is likely to exert a detrimental effect on the locomotor and cognitive performance of healthy young seniors, but that the interference pattern may vary depending on the combination of tasks involved. Therefore, given the potential impact of task difficulty on performance, it may be advisable for individuals over 55 years old, despite their good physical and cognitive health, to perform these complex tasks in a “single-task” manner to ensure optimal task performance.

### Impact of Complexity Level of Locomotor and Cognitive Tasks on DT Abilities

In addition to confirming the presence of DT interference in young seniors, this study also highlights the influence of locomotor and cognitive task complexity on DT abilities. Changes in the magnitude of DTC were observed when participants encountered more complex locomotor and cognitive tasks. Whereas locomotor task complexity level appears to have affected only cognitive DTC, cognitive task complexity impacted both locomotor and cognitive DTC. Other studies have already demonstrated the influence of locomotor task complexity in older adults on locomotor or cognitive DTC, during non-representative of daily life locomotor tasks (Agmon et al., 2014; Plummer-D’Amato et al., 2012). It is well known that performing more complex locomotor tasks leads to higher DTC. According to neuropsychological theories, this observation may be explained by a greater need of attention to perform complex locomotor tasks. In the present study, the need to adjust the trajectory to avoid virtual agent undoubtedly heightened the attentional demand for participants, thus elucidating the impact of the locomotor task complexity on DT performance. As emphasized by several authors (Al-Yahya et al., 2011; Beauchet et al., 2005; Goh et al., 2021), the nature of the cognitive task proposed can significantly impact DT performance. In the present study, the simple cognitive task used can be categorized as a memory task, which solely required the retention of certain information. In contrast, the complex cognitive task involves mental tracking, requiring the retention and manipulation of information throughout the task, thereby requiring a greater allocation of attentional resources. All the observations highlight the importance of task selection while exploring locomotor and cognitive DT abilities. So, for a more comprehensive assessment of individual DT abilities, it seems crucial to utilize scenarios that closely resemble everyday life situations.

### Relationship Between Age and DTC in Healthy Young Seniors

Age-related effects on DT abilities have already been demonstrated in several studies comparing young and older participants (Goh et al., 2021; Nobrego-Sousa et al., 2020). The present study stands out for its targeted sample, as we recruited a group of young seniors (median age: 64) with no locomotor or cognitive impairments. Age-related changes affecting the neural circuits responsible for walking may lead to a reduction in the automatic control process, among other effects (VanSwearingen & Studenski, 2014). It has been shown that to preserve ST and DT locomotor performance, despite the onset of the aging process, young seniors require greater use of their executive resources (evidenced by increased prefrontal activation) compared to younger individuals (Nóbrega-Sousa et al., 2020). However, this compensatory mechanism seems to become inefficient around the age of 70. This phenomenon may explain the observed correlation between age and locomotion DT ability in the present study. In contrast to locomotor DTC, no correlation was observed between age and cognitive DTC, suggesting that, within this specific group of participants, age does not seem to affect cognitive DTC used as measured by the tasks used in this experimental study. It remains possible that more complex or demanding tasks might have revealed an age-related effect. In the present study, the maintenance of cognitive performance may be explained by the concept of cognitive reserve, defined “as a property of the brain that allows for cognitive performance that is better than expected given the degree of life-course related brain changes” (Stern et al., 2023). Vallesi (2016) previously noted that, in healthy older adults, cognitive reserve - quantified by years of education - served as a more accurate predictor of cognitive DTC than either age or general cognitive abilities assessed by the MoCA (Vallesi, 2016). In the present study, recruiting participants through a university list may have minimized the potential impact of age, given a relatively uniform level of cognitive reserve across the group.

## Study Limitations

To explore dual-task ability, we utilized a VR setup connected to an omnidirectional platform, offering a controlled environment with certain limitations. Differences in walking pattern and adaptation to speed have been noted between walking on an omnidirectional platform versus overground (Soni & Lamontagne, 2020). Consequently, the newly learned walking on the omnidirectional platform may request more executive control than overground walking. To minimize the potential effect of a learning phase related to the locomotor pattern imposed using the omnidirectional platform, participants underwent a familiarization period at the end of the first session and again at the beginning of the second visit. Furthermore, DTC were calculated using ST and DT performed with the same equipment.

Despite employing various recruitment methods, Université Laval’s mailing list was the primary source for participant recruitment. Consequently, the majority of recruited participants had completed university-level education, which influenced their cognitive reserve and thus explained the lack of age-related effect on cognitive DTC. The inclusion of participants with a more diverse range of educational backgrounds may lead to increased cost for the dual task. However, the homogeneity of the sample may also be considered a limitation, as it could have reduced the variability in the metrics measured. A more heterogeneous sample might have increased this variance, potentially making it harder to detect differences when they exist.

## Conclusion

The present study investigated DT ability among young seniors during a transitional phase characterized by age-related neurological changes without significant observable behavioral changes in daily activities. During the execution of cognitive-locomotor DT representative of daily life, participants aged between 55 and 75 exhibited locomotor and cognitive DTC. These DTC were notably influenced by the complexity levels of both the locomotor and cognitive tasks, highlighting the importance of employing ecological scenarios to accurately portray cognitive-locomotor activities in everyday life. Moreover, this protocol highlighted an age-related effect of locomotor DTC, but not for cognitive DTC. Such an experimental protocol holds the potential to elucidate the early decline in executive functions, which seems to affect the performance of everyday activities from the onset of the aging process. Consequently, future longitudinal studies are imperative to further explore these interactions.

## Data Availability

Raw data supporting the conclusion of this article are available by the authors upon agreement on the terms of data use and publication of results.*
